# Effect of telmisartan on the expression of adiponectin receptors and nicotinamide adenine dinucleotide phosphate oxidase in the heart and aorta in type 2 diabetic rats

**DOI:** 10.1186/1475-2840-11-94

**Published:** 2012-08-08

**Authors:** Zhixin Guo, Rong Zhang, Jiawei Li, Guojun Xu

**Affiliations:** 1 Department of Endocrinology, Second Hospital, Shanxi Medical University, 382 Wuyi Road, Taiyuan, Shanxi, 030001, P.R. China

**Keywords:** Telmisartan, Adiponectin receptor, NADPH oxidase, Type 2 diabetic, Cardiac, Aorta

## Abstract

**Background:**

Diabetic cardiovascular disease is associated with decreased adiponectin and increased oxidative stress. This study investigated the effect of telmisartan on the expression of adiponectin receptor 2 (adipoR2) and nicotinamide adenine dinucleotide phosphate (NADPH) oxidase subunits in the heart and the expression of adiponectin receptor 1 (adipoR1) in aorta in type 2 diabetic rats.

**Methods:**

Type 2 diabetes was induced by high-fat and high-sugar diet and intraperitoneal injection of a low dose of streptozotocin (STZ). Heart function, adipoR2, p22phox, NOX4, glucose transporter 4(GLUT4), monocyte chemoattractant protein-1(MCP-1) and connective tissue growth factor (CTGF)in the heart, and adipoR1, MCP-1 and nuclear factor kappa B (NF-κB) in aorta were analyzed in controls and diabetic rats treated with or without telmisartan (5mg/kg/d) by gavage for 12 weeks.

**Results:**

Heart function, plasma and myocardial adiponectin levels, the expression of myocardial adipoR2 and GLUT4 were significantly decreased in diabetic rats (P <0.05). The expression of myocardial p22phox, NOX4, MCP-1, and CTGF was significantly increased in diabetic rats (P <0.05). The expression of adipoR1 was decreased and the expression of MCP-1 and NF-κB was increased in the abdominal aorta in diabetic rats (P <0.05). Telmisartan treatment significantly attenuated these changes in diabetic rats (P <0.05).

**Conclusions:**

Our results suggest that telmisartan upregulates the expression of myocardial adiponectin, its receptor 2 and GLUT4. Simultaneously, it downregulates the expression of myocardial p22phox, NOX4, MCP-1, and CTGF, contributing so to the improvement of heart function in diabetic rats. Telmisartan also induces a protective role on the vascular system by upregulating the expression of adipoR1 and downregulating the expression of MCP-1 and NF-κB in the abdominal aorta in diabetic rats.

## Introduction

Cardiovascular disease is one of the major complications of diabetes, resulting in a high percentage of morbidity and mortality and producing significant costs for the healthcare system [1]. Increased fatty acid oxidation and decreased glucose metabolism contribute to the development of diabetic cardiomyopathy and can decrease the ability of the heart to withstand an ischemic insult [2].

Adiponectin is an adipocyte-derived protein with anti-inflammatory, anti-diabetic and anti-atherogenic properties [3]. Adiponectin is also synthesized and secreted by human and murine cardiomyocytes. Local production of adiponectin by cardiomyocytes might have important functions in the regulation of the cardiac function and/or metabolism by autocrine and/or paracrine [4].

There are two types of adiponectin receptors, adiponectin receptor type 1 (adipoR1) and adiponectin receptor type 2 (adipoR2). They serve as receptors for globular and full-length adiponectin, and mediate increased AMP kinase and PPAR-alpha ligand activities, as well as fatty-acid oxidation and glucose uptake by adiponectin. Adiponectin receptor type 1 and adiponectin receptor type 2 are not only expressed in skeletal muscle and liver, but also in heart and kidney [5]. Our previous study showed that the expression of myocardial adipoR1 was significantly decreased in type 2 diabetic rats [6]. It is unknown whether the expression of adipoR2 in the heart and the expression of adipoR1 in aorta are also changed in type 2 diabetic rat.

Oxidative stress has been suggested to be involved in the development and progression of diabetes-induced cardiomyopathy [7]. Activation of nicotinamide adenine dinucleotide phosphate (NADPH) oxidase seems to be relevant to the elevated oxidative stress in diabetes [8]. NAD(P)H oxidase consists of membrane-associated subunits (gp91phox and p22phox) and cytosolic subunits (p47phox, p40phox, p67phox and Rac) [9]. Nox4 is one of homologues of gp91phox/Nox2 [10]. Phagocytic NADPH oxidase largely depends on regulation by cytosolic subunits but not Nox4 for which no cytosolic subunits are required. Nox4 isoform is expressed in a wide variety of organs, including the heart [11] and is a major source of oxidative stress in the failing heart [12]. It was reported that the expression of p22phox and Nox4 in the heart of diabetic mice and rats [13,14] and the expression of p47phox in diabetic rat femoral arteries [15] were significantly increased.

Telmisartan, a unique angiotensin II receptor antagonist with selective peroxisome proliferator-activated receptor gamma(PPARgamma)-modulating activity, functioned as a partial agonist of PPARgamma and achieved 25-30% of maximal receptor activation attained with conventional PPARgamma ligands [16,17]. Telmisartan increased plasma adiponectin level in hypertensive patients with type 2 diabetes [18] and also stimulated adiponectin protein expression in murine 3T3-L1 adipocytes [19]. Telmisartan normalizes vascular dysfunction and reduces platelet activation in diabetic rats [20]. Our previous study showed that telmisartan treatment significantly attenuated the decreased expression of myocardial adipoR1 in diabetic rats [6]. It is unknown whether the expression of adipoR2 and NADPH oxidase subunits in the heart and the expression of adipoR1 in aorta are changed by telmisartane treatment in type 2 diabetic rats.

This study was aimed: 1) to explore the expression of adipoR2 in the heart and the expression of adipoR1 in aorta in type 2 diabetic rats induced by high-fat and high-sugar diet and intraperitoneal injection of a low dose of streptozotocin (STZ); 2) to investigate the effect of telmisartan on the expression of adipoR2 and NADPH oxidase subunits in the heart and the expression of adipoR1 in aorta in type 2 diabetic rats.

## Materials and methods

### Induction of diabetes

Thirty-six male Wistar rats weighing 140-180g, purchased from Physiological Laboratory of Shanxi Medical University (Taiyuan, Shanxi, China), were used in the study. All rats were housed in a temperature-controlled room (22-24^o^C) and kept on a 12 hour light/dark cycle. All animals received humane care in accordance with the principles of the Chinese Council on Animal Care. After two week’s adaptation, all rats were randomly divided into 2 groups: control (C, n = 10) and diabetic (n = 26). Control rats were fed with standard rat chow. Diabetic rats were fed with high-fat chow (ingredients: 10% refined lard, 20% sucrose, 2% cholesterol, 1% sodium cholate and 67% common food), which were provided by Animal Experimental Centre of Shanxi Medical University. Four weeks later, diabetic rats were given the peritoneal injection of a low dose of streptozotocin (30 mg/kg body weight; Sigma, St. Louis, MO, USA) [21], while the control group was given equivalent volume of citric acid buffer. After one week of STZ injection, fasting plasma glucose (FPG) was tested and the rats with FPG ≥7.8 mmol/L and insulin resistance were considered to be diabetic (*n* = 20) [21]. Diabetic rats were again randomly divided into diabetic (D, n = 10) and diabetic treated (DT, n = 10). Telmisartan (5mg/kg/d, Boehringer Ingelheim pharmaceutical company, Germany) [22] was administrated to diabetic treated rats by gavage for 12 weeks. The equivalent volume of normal sodium was administrated to control and diabetic rats by gavage for 12 weeks. Subsequently, blood samples were collected every 2 weeks. Blood was centrifuged at 10,000 g for 45 minutes, plasma was collected and plasma glucose levels were measured. At termination, 1 day before the experiments were finished, all animals were fasted for 12-14 h and then were anesthetized with an intraperitoneal injection of 10% chloral hydrate (0.3mL/100g body weight). The maximum descent-speed of pressure in isovolumetric relaxation period in the left ventricle (-dp/dtmax) and the maximum ascendent-speed of pressure in isovolumic contraction period in the left ventricle (+dp/dtmax) were measured by carotid artery cannula. The rats were sacrificed after blood sample had been withdrawn from abdominal cardinal vein. The blood sample was centrifuged and plasma aliquots were stored at –80°C until assays were done. The heart was immediately taken out of thoracic cavity and the abdominal aorta was immediately taken out of abdominal cavity after the rat was sacrificed. The heart and the abdominal aorta were rinsed with normal sodium and dried by filter-paper and then the heart was weighed. The ratio of heart weight to body weight was calculated. The apex of heart and a part of abdominal aorta were fixed in 10% neutral buffered formalin and processed for histological analysis. The rest part of cardiac ventricle and abdominal aorta were immediately thrown into the liquid nitrogen and then stored in the -70°C refrigerator until analyses were carried out.

### Measurement of cardiac function

Following anesthesia with an intraperitoneal injection of 10% chloral hydrate (0.3ml/100g), the neck skin was cut open and the right common carotid artery was fully exposed. A micromanometer-tipped catheter was inserted into the left ventricle through the right common carotid artery for measurement of left ventricular pressure. Left ventricular end-diastolic pressure (LVEDP) and the maximal rate of rise and decline of ventricular pressure (±dp/dt[max]) were obtained by BL-410 Bio-signal analysis system (Chengdu TME Technology Co., Ltd, Sichuan, China).

### Plasma analytical procedures

The plasma glucose level was measured by the glucose oxidase method using an autoanalyzer (Beckman Instruments, USA). Plasma cholesterol, triglyceride and free fatty acid were measured colorimetrically by using commercially available kit (SiRuiKe Biotechnology Co., Ltd, Shanghai, China). Plasma adiponectin was measured by using a commercially available ELISA kit (Westang Biotechnology Co., Ltd, Shanghai, China). Plasma insulin was measured by using a commercially available radioimmunoassay kit (China Institute of Atomic Energy, Beijing, China). Insulin sensitivity index (ISI) was calculated as the following formula: $\text{ISI}=\text{In}\left[\frac{1}{\left(\text{FPG}\times \text{FINS}\right)}\right]$.

### Determination of cardiac adiponectin

Frozen heart tissue was pulverized and homogenized at 4°C in cold buffer (20 mM Tris-HCl, pH 7.5, 50 mM 2-mercaptoethanol, 5 mM EGTA, 2 mM EDTA, 1 mM PMSF, 10 mM NaF, 25 μg/ml leupeptin, 2 μg/ml aprotinin) and then centrifuged at 1500 g for 5 minutes at 4°C. The supernatant was collected and stored at –80°C until analyses were conducted. The protein content of the samples was measured using the Bradford protein assay [23] with the use of bovine serum albumin as a standard. Cardiac adiponectin was measured using a commercially available enzyme-linked immunosorbent assay (ELISA) kit (Westang Biotechnology Co., Ltd, Shanghai, China).

### Morphologic study

Tissues fixed in 10% buffered formalin were embedded in paraffin, sectioned at 4 μm and stained with hematoxylin and eosin (HE) and Masson’s trichrome for light microscopic morphologic study.

### Immunohistochemical analysis of adipoR1, adipoR2, CTGF, MCP-1 and NF-κB

Ventricular samples and abdominal aorta were immediately fixed in 10% neutral buffered formalin overnight and embedded in paraffin. Paraffin embedded tissue blocks were sectioned at 3 μm and sections were mounted on positively charged slides. The slides were deparaffinized, rehydrated, blocked with 3% hydrogen peroxide [to block endogenous peroxidase activity, washed with phosphate-buffered saline (PBS)], and blocked with 5% normal goat serum in PBS for 30 min. The slides were subsequently incubated with primary rabbit polyclonal adiponectin receptor type 1 (1:200) and type 2 (1:400) antibody (Beijing Biosynthesis Biotechnology Co., Ltd, Beijing, China), connective tissue growth factor antibody (1:100, Wuhan Boster Biological Engineering Company Limited, Hubei, China ), MCP-1 antibody (1:100,Beijing Biosynthesis Biotechnology Co., Ltd, Beijing, China) and NF-κB antibody(1:50,Zhongshan Goldenbridge Biotechnology Co., Ltd, Beijing, China)in PBS containing 1% normal goat serum overnight at 4°C. The primary antibody was rinsed off with PBS, and the sections were incubated with biotin labeling goat anti-rabbit secondary antibodies (1:100, Zhongshan Goldenbridge Biotechnology Co., Ltd, Beijing, China) for 30 min. After three washing steps in PBS were completed, the sections were stained using horseradish enzyme labeling strepto-avidin solution (1:100, Zhongshan Goldenbridge Biotechnology Co., Ltd, Beijing, China) for 10 min, washed with PBS, coloured with 3, 3′-diaminobenzidine (DAB), and washed with distilled water. The sections were counterstained using hematoxylin, washed in running water, dehydrated in increasing grades of alcohol, and cleared in xylene before being mounted in resinous mounting medium with coverslips. Some sections incubated with nonspecific rabbit immunoglobulins (IgG) served as negative controls. Quantification was performed with the observer blinded to details. With the use of high power microscope, all slides were observed and photographed. The average of five fields under microscope (×400) in each slice were randomly selected, and the positive stained intensity or the average value of gray scale which had an inverse proportion to the positive stained intensity was quantified by using BI-2000 color image processing system (Chengdu TME Technology Co., Ltd, Sichuan, China).

### Semi-quantitative reverse transcription-PCR (RT-PCR) analysis for adipoR, MCP-1 and GLUT4

Total RNA extraction was performed using Trizol reagent (Invitrogen, Carlsbad, California, USA) according to the manufacturer’s recommendations. Primer sequences were described in Table 1. The PCR reaction was initiated by 4-minute incubation at 94°C, 32 cycles for denaturation at 94°C for 20 seconds, annealing at 54-56°C for 30 seconds, and extension at 72°C for 30 seconds, terminated after a 10-minute extension at 72°C. Ten microliters of each PCR reaction mixture were electrophoresed in a 1.5% agarose gel and bands were visualized by ethidium bromide staining. The density of the DNA bands of the PCR products was analyzed by using the software (Tianjin DiDe Technology Co., Ltd, Tianjin, China).

**Table 1:** Primers used in semi-quantitative reverse transcription-PCR

**Gene symbol**	**primer (5′→3′)**	**Product size (bp)**
AdipoR1		
Sense	5′-TCTTCCTCATGGCTGTGATG-3′	190bp
Antisense	5′-TCTAGGCCGTAACGGAATTC-3′	
AdipoR2		
Sense	5′-ACCCACAACCTTGCTTCATC-3′	233bp
Antisense	5′-GCTAGCCATGAGCATTAGCC-3′	
GLUT4		
Sense	5′-GATGCCGTCGGGTTTCCAGCA-3′	233bp
Antisense	5′-TGAGGGTGCCTTGTGGGATGG-3′	
MCP-1		
Sense	5′-CTGTCTCAGCCAGATGCAGTT-3′	147bp
Antisense	5′-GAGCTTGGTGACAAATACTACA-3′	
GAPDH		
Forward	5′-TGAACGGGAAGCTCACTGG-3′	307bp
Reverse	5′-TCCACCACCCTGTTGCTGTA-3′	

### Real-time fluorescence quantitative PCR analysis for p22phox and NOX4

Extracted RNA and synthesized cDNA were described above. Real-time quantitative polymerase chain reaction was performed with 50 μl reaction volumes containing 25μl 2× FastStart Universal SYBR Green Master Mix (Roche Ltd., Basel, Switzerland), 1μl 0.3 μM of each primer, 5 μl of cDNA template, and 19 μl deionized water. Primer sequences are described in Table 2. PCR amplifications were done on a real-time fluorescence quantitative PCR detection system (Xi’an Tianlong Science and Technology Co., Ltd,Xi’an, China) using the following parameters: initial denaturation of 95°C for 10 min, followed by 40 cycles for denaturation at 94°C for 30 seconds, annealing at 58°C for 30 seconds, and extension at 72°C for 45 seconds. Relative gene expression levels were quantified using the comparative ΔCt (cycle threshold) method. This method normalized Ct values of the detected gene to the average of that of the housekeeping genes and calculated the relative expression values as fold changes of the control. Ct values were extracted using the software. Data analysis was performed using the 2^-ΔΔCt^ method [24].

**Table 2:** Primers used in real-time fluorescence quantitative PCR

**Gene symbol**	**primer (5′→3′)**	**Product size (bp)**
NOX4		
Forward	5′-TAGCTGCCCACTTGGTGAACG-3′	245bp
Reverse	5′-TGTAACCATGAGGAACAATACCACC-3′	
p22phox		
Forward	5′-CTCTATTGTTGCAGGAGTGC-3′	457bp
Reverse	5′-TCACACGACCTCATCTGTCAC-3′	
β-actin		
Forward	5′-GTCAGGTCATCACTATCGGCAAT-3′	147bp
Reverse	5′-AGAGGTCTTTACGGATGTCAACGT-3′	

### Statistical analysis

Data are expressed as mean ± S.E.M. Statistical analysis was performed by one –way ANOVA followed by Tukey’s post hoc test. P <0.05 was considered statistically significant.

## Results

### Animal characteristics

Plasma glucose levels were significantly increased in diabetic rats compared to controls. Telmisartan treatment slightly but significantly reduced the plasma glucose level in diabetic rats. The levels of plasma insulin, total cholesterol, triglycerides and free fatty acid were significantly increased in diabetic rats compared to controls and telmisartan treatment prevented these changes in diabetic rats. Insulin sensitivity index (ISI) were significantly reduced in diabetic rats compared to controls and telmisartan treatment significantly increased the insulin sensitivity index in diabetic rats. The ratio of heart weight to body weight, an index of cardiac hypertrophy, was significantly increased in diabetic rats compared to controls and telmisartan treatment significantly decreased the ratio of heart weight to body weight in diabetic rats (See table one in reference [6], a paper we previously published. The samples we used in this paper were the same as in reference [6]).

### Histological changes

**Figure 1:**
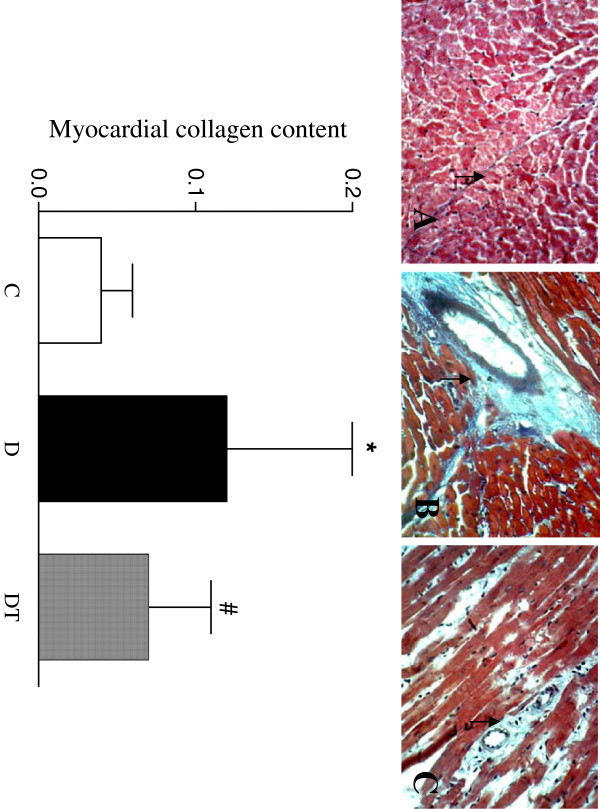
**Top panel: representative slides showing Masson's trichrome staining (Myocardial collagen stained in blue as shown by arrow) in the myocardium. **Slides **A**, **B**, **C **represent control, diabetic and diabetic treated with telmisartan, respectively. Amplifications × 400. Bottom: bar graph shows quantitative analysis of myocardial collagen content in control and diabetic rats treated with or without telmisartan. Control (C), diabetic (D), diabetic treated (DT). Data are expressed as mean ± S.E.M (n = 10 per group). * P <0.05 different from control, ^#^ P <0.05 different from diabetic.

The results of the myocardium stained by Masson’s trichrome and observed through the light microscope were as following: The myocardial cells lined up in order, the spherical or oval cellular nucleus was in the same size and was stained dark and in uniformity, and the cytoplasm was stained red and in uniformity in control rats. The myocardial cells lined up in disorder, the cellular nucleus was in irregular size, breakdown and lost, the myocardial fiber was broken, the boundary between cells was unclear, and collagen fiber was increased in diabetic rats. The pathological changes were obviously improved in diabetic rats treated with telmisartan. The myocardial collagen content was increased in diabetic rats and was decreased in diabetic rats treated with telmisartan (Figure 1).

**Figure 2:**
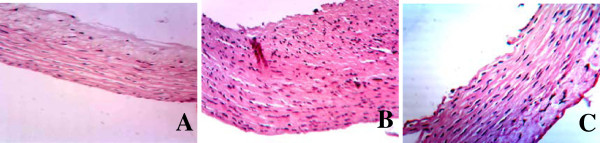
**Representative slides showing HE staining in the abdominal aorta of rats. **Slides **A**, **B**, **C **represent control, diabetic and diabetic treated with telmisartan, respectively. Amplifications × 200.

The results of the aorta stained by hematoxylin and eosin (HE) and observed through the light microscope were as following: the intima of aorta was smooth, all level cells lined up in order, endothelial cells were applanate and clung to the smooth and straight internal elastic lamina, and medial elastic lamina lined up in parallel with smooth muscle cell orderly and alternately in control rats. Endothelial cells were broken off and the shallow smooth muscle cells in medial elastic lamina were proliferated and lined up in disorder in diabetic rats. The pathological changes of aorta were obviously improved in diabetic rats treated with telmisartan (Figure 2).

### Heart function

Compared to controls, +dp/dtmax and -dp/dtmax were significantly reduced and LVEDP was significantly increased in diabetic rats, indicating that the heart function was significantly decreased in diabetic rats. Telmisartan treatment attenuated these changes in diabetic rats (See table two in reference [6], a paper we previously published. The samples we used in this paper were the same as in reference [6]).

### Immunohistochemical assay

**Figure 3:**
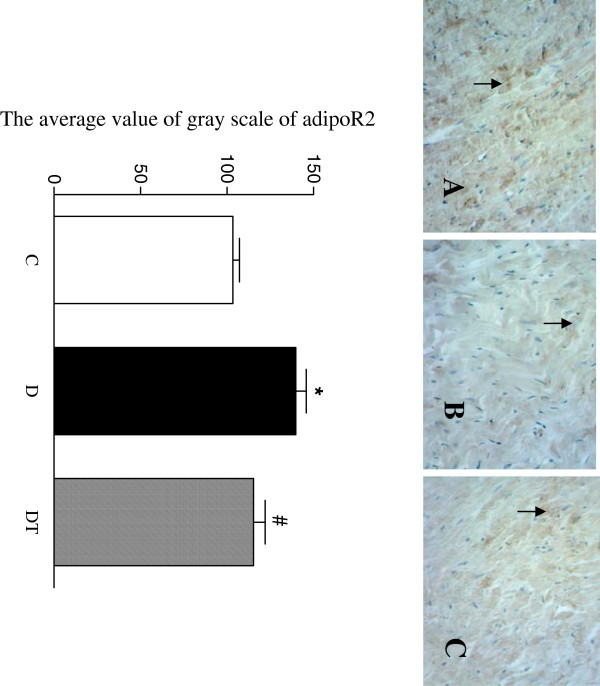
**Top panel: representative slides showing immunohistochemical staining of AdipoR2 (stained in brown as shown by arrow) in the myocardium. **Slides **A**, **B**, **C **represent control, diabetic and diabetic treated with telmisartan, respectively. Amplifications × 400. Bottom: bar graph shows quantitative analysis of myocardial adipoR2 expression in control and diabetic rats treated with or without telmisartan. The adipoR2 expression was quantified using the average value of gray scale which had an inverse proportion to the positive stained intensity. Control (C), diabetic (D), diabetic treated (DT). Data are expressed as mean ± S.E.M (n = 10 per group). * P <0.05 different from control, ^#^ P <0.05 different from diabetic.

The yellow positive staining of adipoR2 was mainly located in myocardial cellular member and cytoplasm. The adipoR2 expression was quantified using the average value of gray scale which had an inverse proportion to the positive stained intensity. The average value of gray scale of adipoR2 was significantly increased in the heart in diabetic rats compared to controls, indicating that the positive stained intensity of adipoR2 was significantly decreased in the heart in diabetic rats compared to controls. Telmisartan treatment significantly decreased the average value of gray scale of adiponectin receptor 2 in the heart in diabetic rats, indicating that telmisartan treatment significantly increased the positive stained intensity of adipoR2 in the heart in diabetic rats (Figure 3).

**Figure 4:**
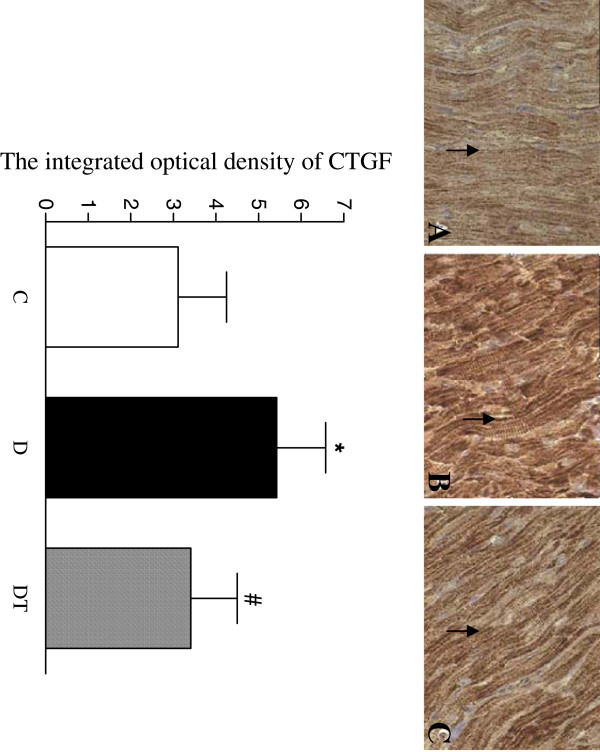
**Top panel: representative slides showing immunohistochemical staining of CTGF (stained in brown as shown by arrow) in the myocardium. **Slides **A**, **B**, **C **represent control, diabetic and diabetic treated with telmisartan, respectively. Amplifications × 400. Bottom: bar graph shows quantitative analysis of myocardial CTGF expression in control and diabetic rats treated with or without telmisartan. Control (C), diabetic (D), diabetic treated (DT). Data are expressed as mean ± S.E.M (n = 10 per group). * P <0.05 different from control, ^#^ P <0.05 different from diabetic.

The yellow positive staining of CTGF was mainly located in myocardial cellular cytoplasm. The integrated optical density(IOD)of positive staining of CTGF was significantly increased in the heart in diabetic rats compared to controls. Telmisartan treatment significantly decreased the integrated optical density (IOD) of positive staining of CTGF in the heart in diabetic rats (Figure 4).

**Figure 5:**
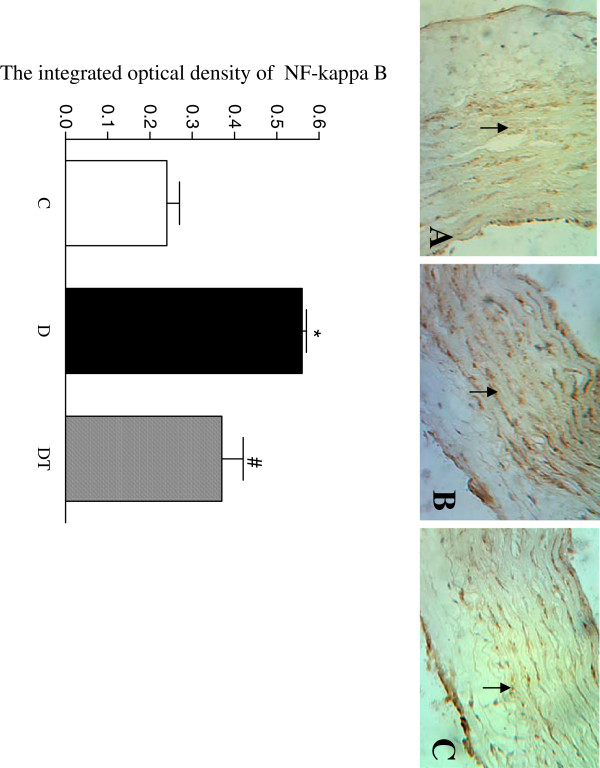
**Top panel: representative slides showing immunohistochemical staining of NF-κB (stained in brown as shown by arrow) in abdominal aorta. **Slides **A**, **B**, **C **represent control, diabetic and diabetic treated with telmisartan, respectively. Amplifications × 200. Bottom: bar graph shows quantitative analysis of NF-κB expression in aorta in control and diabetic rats treated with or without telmisartan. Control (C), diabetic (D), diabetic treated (DT). Data are expressed as mean ± S.E.M (n = 10 per group). * P <0.05 different from control, ^#^ P <0.05 different from diabetic.

**Figure 6:**
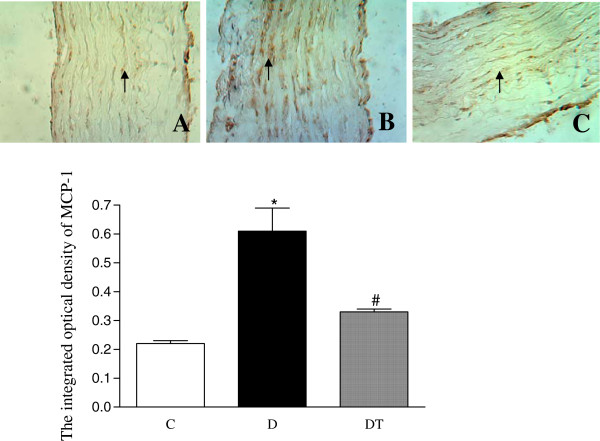
**Top panel: representative slides showing immunohistochemical staining of MCP-1 (stained in brown as shown by arrow) in abdominal aorta. **Slides **A**, **B**, **C **represent control, diabetic and diabetic treated with telmisartan, respectively. Amplifications × 200. Bottom: bar graph shows quantitative analysis of MCP-1 expression in aorta in control and diabetic rats treated with or without telmisartan. Control (C), diabetic (D), diabetic treated (DT). Data are expressed as mean ± S.E.M (n = 10 per group). * P <0.05 different from control, ^#^ P <0.05 different from diabetic.

The yellow positive staining of NF-κB was mainly located in cellular nucleus and the yellow positive staining of MCP-1 was mainly located in cellular cytoplasm in abdominal aorta. The integrated optical density (IOD) of positive staining of NF-κB and MCP-1 was significantly increased in aorta in diabetic rats compared to controls. Telmisartan treatment significantly decreased the integrated optical density (IOD) of positive staining of NF-κB and MCP-1 in aorta in diabetic rats (Figures 5 and 6).

### Plasma and myocardial adiponectin levels

Plasma and myocardial adiponectin levels were significantly decreased in diabetic rats compared to controls. Telmisartan treatment significantly increased plasma and myocardial adiponectin levels in diabetic rats (See Table two in reference [6], a paper we previously published. The samples we used in this paper were the same as in reference [6]).

### Myocardial mRNA expression of adiponectin receptor 2, GLUT4, MCP-1, NOX4 and p22phox

**Figure 7:**
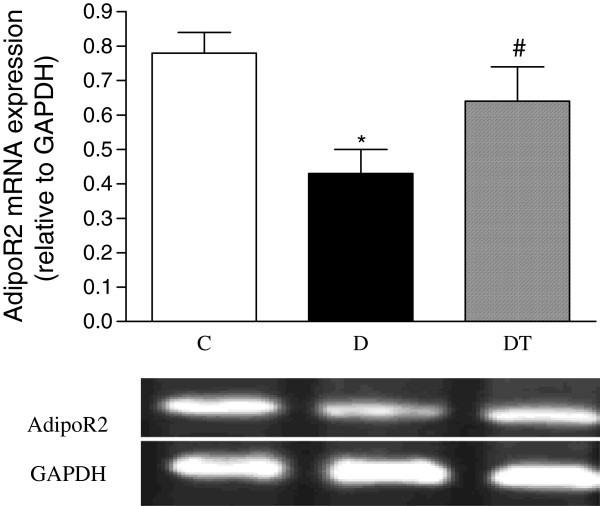
**RT-PCR analysis of myocardial mRNA expression of adipoR2 in control and diabetic rats treated with or without telmisartan. **Mean band density was normalized relative to GAPDH. Control (C), diabetic (D), diabetic treated (DT). Data are expressed as mean ± S.E.M (n = 10 per group). * P <0.05 different from control, ^#^ P <0.05 different from diabetic.

**Figure 8:**
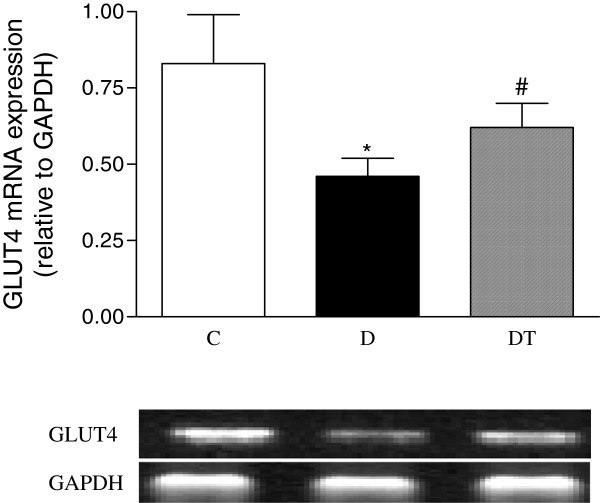
**RT-PCR analysis of myocardial mRNA expression of GLUT4 in control and diabetic rats treated with or without telmisartan. **Mean band density was normalized relative to GAPDH. Control (C), diabetic (D), diabetic treated (DT). Data are expressed as mean ± S.E.M (n = 10 per group). * P <0.05 different from control, ^#^ P <0.05 different from diabetic.

The mRNA expression of myocardial adiponectin receptor 2 and GLUT4 was significantly reduced in diabetic rats compared to controls. Telmisartan treatment significantly increased the mRNA expression of myocardial adiponectin receptor 2 and GLUT4 in diabetic rats (Figures 7 and 8).

**Figure 9:**
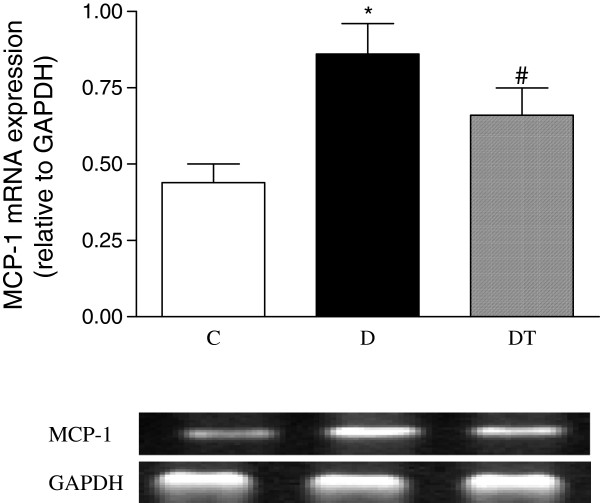
**RT-PCR analysis of myocardial mRNA expression of MCP-1 in control and diabetic rats treated with or without telmisartan. **Mean band density was normalized relative to GAPDH. Control (C), diabetic (D), diabetic treated (DT). Data are expressed as mean ± S.E.M (n = 10 per group). * P <0.05 different from control, ^#^ P <0.05 different from diabetic.

**Figure 10:**
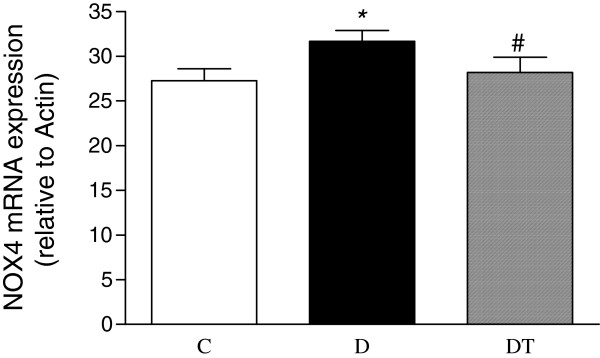
**Real-time fluorescence quantitative PCR analysis of myocardial mRNA expression of NOX4 in control and diabetic rats treated with or without telmisartan. **Relative gene expression levels were normalized relative toβ-actin. Control (C), diabetic (D), diabetic treated (DT). Data are expressed as mean ± S.E.M (n = 10 per group). * P <0.05 different from control, ^#^ P <0.05 different from diabetic.

**Figure 11:**
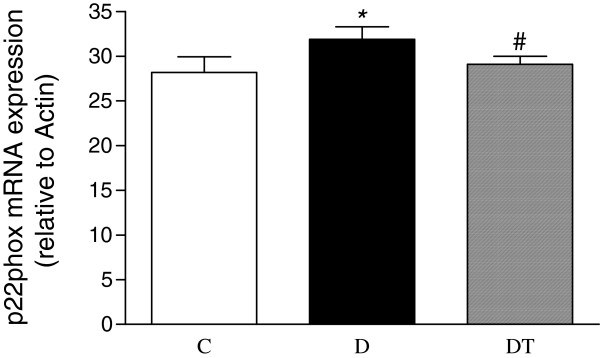
**Real-time fluorescence quantitative PCR analysis of myocardial mRNA expression of p22phox in control and diabetic rats treated with or without telmisartan. **Relative gene expression levels were normalized relative toβ-actin. Control (C), diabetic (D), diabetic treated (DT). Data are expressed as mean ± S.E.M (n = 10 per group). * P <0.05 different from control, ^#^ P <0.05 different from diabetic.

The mRNA expression of myocardial MCP-1, NOX4 and p22phox was significantly increased in diabetic rats compared to controls. Telmisartan treatment significantly reduced the mRNA expression of myocardial MCP-1, NOX4 and p22phox in diabetic rats (Figures 9, 10 and 11).

### The mRNA expression of adiponectin receptor 1 in abdominal aorta

**Figure 12:**
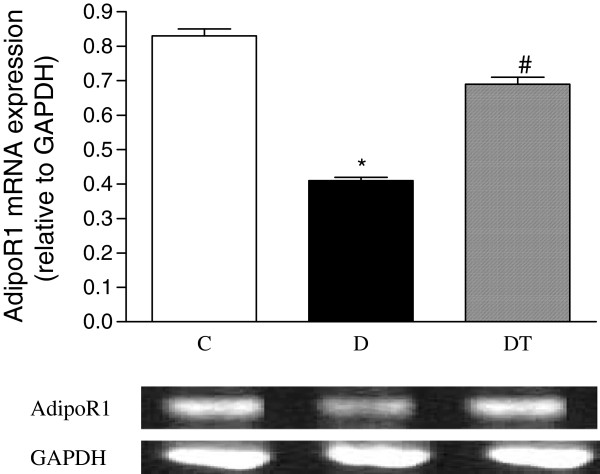
**RT-PCR analysis of mRNA expression of adipoR1 in abdominal aorta in control and diabetic rats treated with or without telmisartan. **Mean band density was normalized relative to GAPDH. Control (C), diabetic (D), diabetic treated (DT). Data are expressed as mean ± S.E.M (n = 10 per group). * P <0.05 different from control, ^#^ P <0.05 different from diabetic.

The mRNA expression of adiponectin receptor 1 in abdominal aorta was significantly reduced in diabetic rats compared to controls. Telmisartan treatment significantly increased the mRNA expression of adiponectin receptor 1 in abdominal aorta in diabetic rats (Figure 12).

## Discussion

This study showed that diabetic rat hearts exhibited increased expression of CTGF and collagen content with concomitant cardiac hypertrophy and fibrosis. Plasma and myocardial adiponectin levels, the mRNA expression of adipoR2 in heart, and the mRNA expression of adipoR1 in aorta were decreased in type 2 diabetic rats. The myocardial mRNA expression of NADPH oxidase subunits, p22phox and Nox4, was increased in type 2 diabetic rats. Telmisartan treatment prevented these changes in diabetic rats.

### Effect of telmisartan on the expression of adiponectin receptors in the heart and aorta in type 2 diabetic rats

Adiponectin exerts its effect through adiponectin receptor 1 and 2, which are both expressed in the heart in vivo [5] and in the cardiomyocyte in vitro [4]. Adiponectin receptors were also expressed in atherosclerotic lesions, macrophages and vascular endothelial cells [5,25]. Overweight patients with coronary artery disease had decreased surface expression of adiponectin receptors in peripheral monocytes [26]. Although the protein expression of adiponectin receptor 1 in coronary arterioles and aortas was similar between control and diabetic mice, the protein expression of adiponectin receptor 2 was significantly reduced in type 2 diabetic mice [27]. Adenovirus-mediated overexpression of AdipoR1 and 2 in vascular endothelial cells significantly enhanced the antiinflammatory effect of adiponectin [28]. Our study showed that the expression of adipoR2 in the heart and the expression of adipoR1 in aorta were decreased in diabetic rats. The decreased adiponectin receptors may be due to hyperinsulinemia since it has been reported that insulin deficiency increased, but insulin replenishment decreased the expression of adipoR1/2 in animals in vivo [29]. The decreased adiponectin and its receptors may lead to adiponectin resistance, which may limit adiponectin to produce its biological effects, especially its protective effect on diabetic heart and aorta. The decreased adiponectin and its receptors may together play a role in the occurrence and progression of cardiomyopathy and atherosclerosis in type 2 diabetic rats. Adiponectin receptors could be modulated by agonists of the nuclear receptors PPARalpha, PPARgamma, and LXR [25]. Our study showed that telmisartan, a unique angiotensin II receptor antagonist with selective PPARgamma-modulating activity [16,17], increased the mRNA expression of AdipoR2 in the heart and the mRNA expression of AdipoR1 in the aorta in diabetic rats, indicating that angiotensin II could down-regulate, while PPARgamma agonist could up-regulate the expression of adiponectin receptors. The results of our study are in agreement with the results of studies [25,30] showing that adiponectin receptors were expressed in adult ventricular cardiomyocytes, atherosclerotic lesions and macrophages, and upregulated by activation of peroxisome proliferator-activated receptor gamma.

Adiponectin can improve both glucose metabolism and insulin resistance [31]. Glucose enters the heart via the facilitative glucose transporters GLUT1 and GLUT4 [32]. Glucose transporter expression in the heart is altered in various pathological states. Our study showed that the mRNA expression of GLUT4 was decreased, indicating that glucose metabolism would be reduced in diabetic rat hearts. It was reported that changes in glucose transporter expression contributed to myocardial dysfunction in diabetes [33]. In addition, GLUT4-deficient mice developed striking cardiac hypertrophy [34]. Normalization of glucose homeostasis by transgenic re-expression of GLUT4 in the skeletal muscle resulted in a reversal of the cardiac pathology in mice heterozygous for GLUT4 ablation [35]. Hence, we propose that the decreased cardiac GLUT4 observed in this study may contribute to the deterioration in heart function and to the cardiac hypertrophy seen in diabetic rats. Our study showed that telmisartan treatment reduces cardiac hypertrophy, improved the heart function, and increased myocardial expression of GLUT4 in diabetic rats. The increased adiponectin and its receptors may partly explain the increased GLUT4, which may contribute to the ameliorated heart function in diabetic rats treated with telmisartan.

### Effect of telmisartan on the expression of inflammatory cytokines in the heart and aorta in type 2 diabetic rats

Heart failure, diabetes, and obesity are recognized as states of chronic inflammation. Inflammatory cytokines may play a role in all three of these conditions [36,37]. Monocyte chemotactic protein-1 (MCP-1) has been shown to be produced and released by the heart and to be increased in failing heart [38,39]. Increased levels of MCP-1 are associated with previously unknown abnormal glucose regulation in patients with acute ST-elevation myocardial infarction [40]. Our study showed that cardiac expression of MCP-1 was increased in diabetic rats. Telmisartan treatment decreased the cardiac expression of MCP-1 in diabetic rats. It was reported that adiponectin suppressed MCP-1 production in lipopolysaccharide-treated 3T3-L1 adipocytes [41]. Oxidative stress increased the level of MCP-1[42]. Therefore, the elevated cardiac MCP-1 may be due to the decreased adiponectin and the increased oxidative stress, and may be involved in the diabetic cardiac deterioration. The increased adiponectin and decreased NADPH oxidase by telmisartan treatment may lead to the decreased inflammation factor MCP-1 in the heart, which may result in the improved heart function in diabetic rats.

This study showed that the expression of MCP-1 and NF-κB was increased in aorta in diabetic rats. Telmisartan treatment significantly reduced the expression of MCP-1 and NF-κB and alleviated the pathological alteration in aorta in diabetic rats, suggesting that telmisartan may lessen the atherosclerotic degree by downregulating the expression of MCP-1 and NF-κB in aorta in diabetic rats. It was reported that adiponectin reduced aortic NF-kappaB expression in ApoE knockout mice and inhibited IκBα phosphorylation and nuclear factor κB protein expression in aorta of type 2 diabetic mice [27,43]. Adenovirus-mediated overexpression of AdipoR significantly enhanced the suppressive effect of adiponectin on NF-kappaB activation in vascular endothelial cells [28]. Therefore, the increased expression of MCP-1 and NF-κB in aorta may be associated with the reduced expression of adiponectin and its receptors.

### Effect of telmisartan on the expression of NADPH oxidase in the heart in type 2 diabetic rats

Oxidative stress has been suggested to be involved in the development and progression of diabetes-induced cardiomyopathy (7). NADPH oxidase has emerged as the main source of ROS in the cardiovascular tissues [44]. It was reported that the expression of NADPH oxidase subunits, p22phox and Nox4 was significantly increased in the heart of diabetic mice and rats [13,14,45,46], indicating that Nox4 was an important source of ROS in the left ventricle and Nox4-derived ROS contribute to cardiomyopathy at early stages of type 1 diabetes [45]. Consistent with these studies, the present study also showed that the expression of myocardial p22phox and Nox4 was significantly increased in type 2 diabetic rats, suggesting that NADPH oxidase might play a crucial role in the development of diabetic cardiomyopathy. It was reported that telmisartan treatment reduced the protein expression of renal and vascular NADPH oxidase subunits in diabetic mice and cardiac NADPH oxidase activity in diabetic rats [47,48]. Our study showed that telmisartan treatment decreased the myocardial mRNA expression of NADPH oxidase subunits, p22phox and Nox4, which may result in the reduced oxidative stress and the improved heart function, in diabetic rats.

## Conclusions

In summary, this study demonstrates that diabetic rat hearts exhibit increased expression of CTGF and collagen content accompanied by cardiac hypertrophy and in creased fibrosis. The plasma and myocardial adiponectin levels and the mRNA expression of myocardial adipoR2 are decreased, which may lead to the decrease in cardiac GLUT 4 and the increase in cardiac MCP-1 in diabetic rats. The cardiac mRNA expression of p22phox and NOX4 is significantly increased, indicating that oxidative stress is increased in the heart in diabetic rats. The decreased mRNA expression of adipoR1 may lead to the increased expression of MCP-1 and NF-κB in aorta in diabetic rats. Telmisartan up-regulates the expression of myocardial adiponectin and its receptor 2 and GLUT4, down-regulates the expression of myocardial p22phox, NOX4, MCP-1, and CTGF, which may contribute to the improvement of heart function in diabetic rats. Telmisartan may also produce the protective role on the vascular by upregulating the expression of adipiR1 and downregulating the expression of MCP-1 and NF-κB in the abdominal aorta in diabetic rats.

## Abbreviations

NADPH, Nicotinamide adenine dinucleotide phosphate; STZ, Streptozotocin; adipoR2, Adiponectin receptor 2; adipoR1, Adiponectin receptor 1; GLUT4, Glucose transporter 4; MCP-1, Monocyte chemoattractant protein-1; CTGF, Connective tissue growth factor; NF-κB, Nuclear factor kappa B; PPARgamma, Peroxisome proliferator-activated receptor gamma; FPG, Fasting plasma glucose; LVEDP, Left ventricular end-diastolic pressure; ISI, Insulin sensitivity index; HE, Hematoxylin and eosin; ELISA, Enzyme-linked immunosorbent assay; PBS, Phosphate-buffered saline; DAB, Diaminobenzidine; IOD, Integrated optical density; GAPDH, Glyceraldehyde-3- phosphate dehydrogenase.

## Competing interests

All Authors declare that they have no competing interests.

## Authors’ contributions

ZG designed the study and drafted the manuscript. RZ carried out Masson’s trichrome staining, detected the expression of adiponectin receptor 2, MCP-1 and GLUT-4 in the heart, and participated in the statistical analysis. JL contributed to the assay of p22phox, NOX4 and CTGF in the heart, and participated in the statistical analysis. GX carried out HE staining, detected the expression of adiponectin receptor 1, MCP-1 and NF-κB in the aorta, and participated in the statistical analysis. All authors read and approved the final manuscript.
